# Propofol-Remifentanil Combination for Management of Electroconvulsive Therapy in a Patient with Neuroleptic Malignant Syndrome

**DOI:** 10.1155/2012/585713

**Published:** 2012-04-03

**Authors:** Modabber Arasteh, Shoaleh Shami, Karim Nasseri

**Affiliations:** ^1^Department of Psychiatry, Faculty of Medicine, Kurdistan University of Medical Sciences, Sanandaj, Iran; ^2^Faculty of Nursing and Midwifery, Kurdistan University of Medical Sciences, Sanandaj, Iran; ^3^Department of Anesthesia, Kurdistan University of Medical Sciences, Beasat Hospital, Sanandaj, Iran

## Abstract

Electroconvulsive therapy can be effective in severe or treatment resistant neuroleptic malignant syndrome patients. Anesthesia and use of muscle relaxant agents for electroconvulsive therapy in such patients may encounter anesthesiologists with specific challenges. This case report describes successful management of anesthesia in 28-year-old male patient undergoing eight electroconvulsive therapy sessions for treatment of neuroleptic malignant syndrome.

## 1. Introduction

Neuroleptic malignant syndrome (NMS) is a rare, unpredictable, and potentially fatal complication of certain antipsychotic medications. The incidence and mortality rates of NMS are approximately 0.07% to 2.2%, and 15%–25%, respectively [1]. Typical characteristics consist of hyperthermia, altered level of conscious, muscle rigidity, catatonia, autonomic dysfunction, and elevation of Creatine Kinase (CK) level. All the previous findings are not necessary for diagnosis [1]. Bromocriptine, dantrolene, and benzodiazepines are effective in the treatment of NMS [2, 3]. Some authors recommend that electroconvulsive therapy (ECT) may be effective if signs and symptoms are resistant to supportive care and pharmacotherapy [4, 5].

There are a known sameness between the clinical symptoms of NMS and malignant hyperthermia (MH) [6]; therefore several reports have incited some controversy regarding the safety of the use of known triggering agents of MH such as succinylcholine in patients with a history of NMS [6–8]. Anesthesia and muscle relaxation for ECT in such patients poses specific challenges to anesthesiologists. This case report describes successful use of propofol-remifentanil without muscle relaxant in a patient undergoing ECT for the treatment of NMS.

## 2. Case Report

A 28-year-old male with a history of bipolar mood disorder and moderate mental retardation was admitted to hospital with increasing body temperature, muscle rigidity, diaphoresis, cognitive impairment, and some degrees of impaired consciousness. He had been taking thioridazine and valproate for a period of 6 months prior to hospital admission. On examination the patient was mute and showed unusual postures characteristic of catatonia. His vital signs were as follows: temperature (38.2°C), blood pressure 131/82 mm Hg, heart rate 114 beats/min, and respiratory rate 18. The CK level was markedly elevated (1245 U/L) other laboratory results, including electrolytes, were slightly elevated. He had several psychiatric hospitalizations, during the last years prior to the present episode, and lived in a chronic mental disorder institute. In family history his monozygotic twin had died with NMS manifestations about one year ago. Antipsychotic drugs were stopped, and diagnosis of NMS was made, because the patient met three major (rigidity, hyperthermia, rising of CK) and three minor criteria (tachycardia, diaphoresis, cognitive impairment) of NMS. After six days because of progressive catatonia and muscular rigidity ECT under anesthesia was considered. 

After preoxygenation with 100% O_2_ via face mask, anesthesia was induced with remifentanil 1.5 *μ*g/kg and propofol 1.5 mg/kg IV slowly. Then the ECT stimulus was applied, and it produced an ensuing seizure. Succinylcholine and any other muscle relaxants were avoided. The doses of propofol and remifentanil remained fixed in all eight sessions of ECT and there was no need for adding other anesthetic or analgesic drugs for inducing anesthesia. Bilateral ECT was administered using an ECT stimulator (brief pulse rate, analog-output ECT: type RF class 1. ARA 121; ARA Co Ltd., Tehran, Iran). The first selected stimulus intensity was 20% that induced a 32-second relatively tense seizure. The stimulus intensity titrated based on previous seizure length and reached 50% in the last session. The range of seizure duration was 24–37 second (mean 27 second). The mean time from inducing anesthesia to returning spontaneous ventilation was 8 minutes [7–10], and patient opened his eyes by commend after 9–14 minutes (mean 11 minutes). The body temperature was measured on several occasions following ECT, at intervals of 3-4 hour. We did not observe any changes in body temperature. After eight ECT sessions that was done three times a week, the psychotic symptoms improved considerably, CK level turned back toward normal ranges slightly, and the patient was discharged from hospital without further complications.

## 3. Discussion

Although NMS and MH result from different pathophysiological mechanisms [1, 9], however, they have some common clinical features. So patients susceptible to NMS may be vulnerable to developing MH. ECT has been used as a treatment for NMS, because it supposedly increases circulating catecholamines, including dopamine, in the central nervous system [2, 3].

Succinylcholine remains the most commonly used muscle relaxant to decrease the strong muscle contractions associated with ECT-induced seizure activity [10]. However, the use of this rapid and short-acting drug can accompany with side effects in patients with history of susceptibility to MH, NMS, and catatonic schizophrenia [11, 12]. And it has been suggested that the use of succinylcholine should be avoided in patients with a history of these diseases [13].

Although nondepolarizing agents has been used successfully in the treatment of NMS, some authors suggest that these drugs are associated with increases in temperature, CK levels, and leukocytosis [14] too, so it may be advisable to avoid administering depolarizing and nondepolarizing muscle relaxants to patients with NMS. Based on this information, we decided to perform ECT using remifentanil-propofol without muscle relaxant in this case. In the sole related case report that we find, Franks et al. [15] performed ECT on an MH susceptible patient using thiamylal without any muscle relaxant, but the patient required physical control during the seizure and experienced severe muscle pain after the procedure.

Remifentanil is an ultra-short-acting opioid with a rapid onset and an ultrashort context sensitive half-time [16]. Propofol has significant myorelaxant properties on pharyngeal and laryngeal structures, so it is the agent of choice for intubation without muscle relaxant [17]. On the other hand, there are some reports that the infusion of propofol and remifentanil slightly decrease both the train-of-four ratio in comparison with the preoperative value [18] and succinylcholine-induced fasciculation [19, 20]. Based on the results reported herein, and the aforementioned pharmacological properties, individuals with NMS may be at providing general anesthesia using remifentanil-propofol boluses without neuromuscular blocking agents and were able to complete sessions of ECT without any associated complications.

## 4. Conclusion

It is likely that combination of remifentanil-propofol without muscle relaxants for ECT in NMS patients is safe and effective.
